# Characterization of physiochemical properties of caveolin-1 from normal and prion-infected human brains

**DOI:** 10.18632/oncotarget.19431

**Published:** 2017-07-21

**Authors:** Xiangzhu Xiao, Pingping Shen, Zerui Wang, Johnny Dang, Alise Adornato, Lewis S. Zou, Zhiqian Dong, Jue Yuan, Jiachun Feng, Li Cui, Wen-Quan Zou

**Affiliations:** ^1^ Department of Pathology, Case Western Reserve University, Cleveland, OH, USA; ^2^ Institute of Neuroscience Center and Neurology Department, The First Hospital of Jilin University, Changchun, Jilin, China; ^3^ State Key Laboratory for Infectious Disease Prevention and Control, National Institute for Viral Disease Control and Prevention, Chinese Center for Disease Control and Prevention, Beijing, China

**Keywords:** caveolin-1, human brain, prion disease, solubility, aggregation, Gerotarget

## Abstract

Caveolin-1 is a major component protein of the caveolae—a type of flask shaped, 50-100 nm, nonclathrin-coated, microdomain present in the plasma membrane of most mammalian cells. Caveolin-1 functions as a scaffolding protein to organize and concentrate signaling molecules within the caveolae, which may be associated with its unique physicochemical properties including oligomerization, acquisition of detergent insolubility, and association with cholesterol. Here we demonstrate that caveolin-1 is detected in all brain areas examined and recovered in both detergent-soluble and -insoluble fractions. Surprisingly, the recovered molecules from the two different fractions share a similar molecular size ranging from 200 to 2,000 kDa, indicated by gel filtration. Furthermore, both soluble and insoluble caveolin-1 molecules generate a proteinase K (PK)-resistant C-terminal core fragment upon the PK-treatment, by removing ˜36 amino acids from the N-terminus of the protein. Although it recognizes caveolin-1 from A431 cell lysate, an antibody against the C-terminus of caveolin-1 fails to detect the brain protein by Western blotting, suggesting that the epitope in the brain caveolin-1 is concealed. No significant differences in the physicochemical properties of caveolin-1 between uninfected and prion-infected brains are observed.

## INTRODUCTION

Caveolae are invaginations of the plasma membrane with a diameter of 60-80 nm, formed by the polymerization of caveolin-1 and a subset of lipid-raft components, including cholesterol and sphingolipids [1]. Caveolae have been implicated in endocytosis, transcytosis, calcium signaling and numerous other signal transduction events in several types of cells including neurons. Caveolin-1 is the only known protein component of the caveolae. It has an unusual membrane topology with N and C termini in the cytoplasm and a long putative hairpin intramembrane domain. Oligomerization and association of caveolin-1 with cholesterol-rich lipid-raft domains are involved in the formation of caveola [1]. Caveolin-1 binds 1 to 2 cholesterol molecules and is also palmitoylated in the C-terminal regions.

A number of human diseases, including prion diseases, appear to involve the caveolae membrane system [2]. PrP^C^ has been found to localize in invaginated caveolae in both fibroblasts and neuronal N2A cells and fractionates with caveolae. Replacement of the GPI anchor of PrP^C^ with a coated pit targeting sequence prevents conversion, suggesting that caveolae localization appears to be necessary for conversion of PrP^C^ to PrP^Sc^; lowering cellular cholesterol, which disperses GPI proteins in the membrane, also inhibits conversion [3, 4]. Moreover, accumulation of PrP^Sc^ may impair many different caveolae functions. Nevertheless, so far, caveolin-1 from human brains has not been fully investigated, especially in prion-infected human brains.

Here we characterize caveolin-1 from prion infected and uninfected human brains. We demonstrate that caveolin-1 is widely present in different brain areas. There are detergent-soluble and insoluble isoforms of caveolin-1 in the human brain. The two isoforms share similar oligomeric state and resistance to PK-digestion. There are no significant differences in the physicochemical properties of caveolin-1 between uninfected and prion-infected brains.

## RESULTS

### Caveolin-1 is widely present in a variety of human brain areas

**Figure 1:**
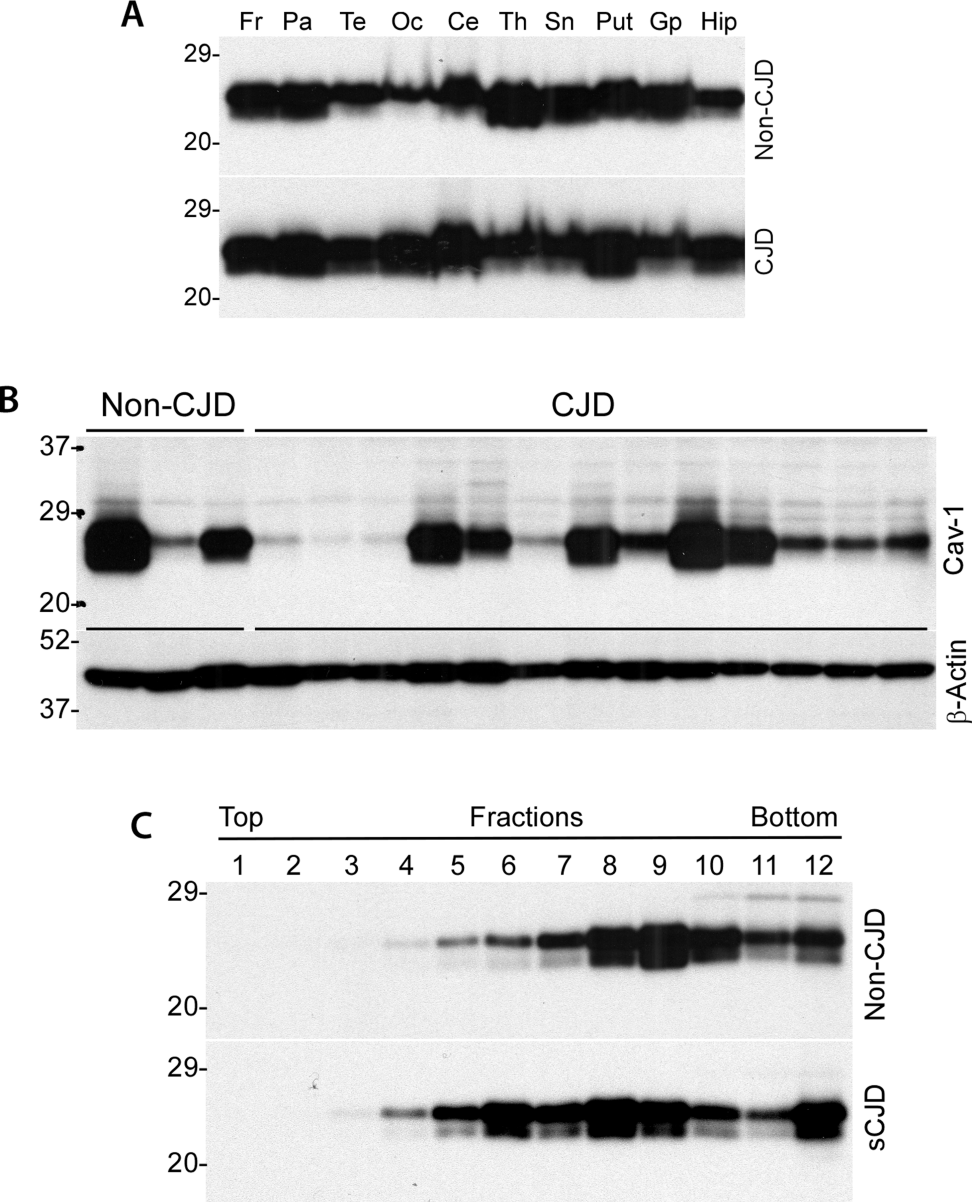
Distribution of caveolin-1 in different brain areas from CJD and non-CJD individuals **A**. Western blotting of caveolin-1 from ten areas were examined including frontal cortex (Fr), parietal cortex (Par), temporal cortex (Te), occipital cortex (Oc), cerebellum (Ce), thalamus (Th), substantial nigra (Sn), putamen (Put), globus pallidus (Gp), and hippocampus (Hip). **B**. Western blotting of caveolin-1 between non-CJD and CJD patients. β-actin was used as to monitor amount of samples loaded in each lane. **C**. Western blotting of caveolin-1 of normal human brain homogenate from non-linear sucrose gradient sedimentation fractions. All Western blots are representative of three independent experiments.

Using Western blot analysis, we first investigated the distribution of caveolin-1 in different brain areas in normal individuals and patients with sporadic Creutzfeldt-Jakob disease (sCJD), the most common form of human prion disease. Ten areas were examined including frontal cortex (Fr), parietal cortex (Par), temporal cortex (Te), occipital cortex (Oc), cerebellum (Ce), thalamus (Th), substantial nigra (Sn), putamen (Put), globus pallidus (Gp), and hippocampus (Hip). Caveolin-1 was detected in all brain areas of both normal and sCJD patients examined (Figure 1A). Two bands migrating at ˜ 24 kDa and ˜22 kDa were observed, which are expected to correspond to α-form and β-form of caveolin-1, respectively. There were substantial variations in the levels of caveolin-1 from one area to another area (Figure 1A) as well as from case to case (Figure 1B). However, there were no significant differences in caveolin-1 levels between non-CJD and sCJD patients (Figure 1B). Using sucrose linear gradients, caveolin-1 from various cell lines has been demonstrated to exist as oligomers at molecular mass of 300-600 kDa [5]. The brain homogenates from both non-CJD and sCJD were subjected to ultracentrifugation with non-linear sucrose gradients from 10-60%. As shown in Figure 1C, caveolin-1 from non-CJD and sCJD brain homogenates was recovered from fractions 4 to 12, predominantly in fractions 8 and 9, similar to the observation reported previously [2]. No significant differences in oligomeric state of caveolin-1 were determined between non-CJD and sCJD.

### There are both detergent-soluble and detergent-insoluble forms of caveolin-1 in human brains

**Figure 2:**
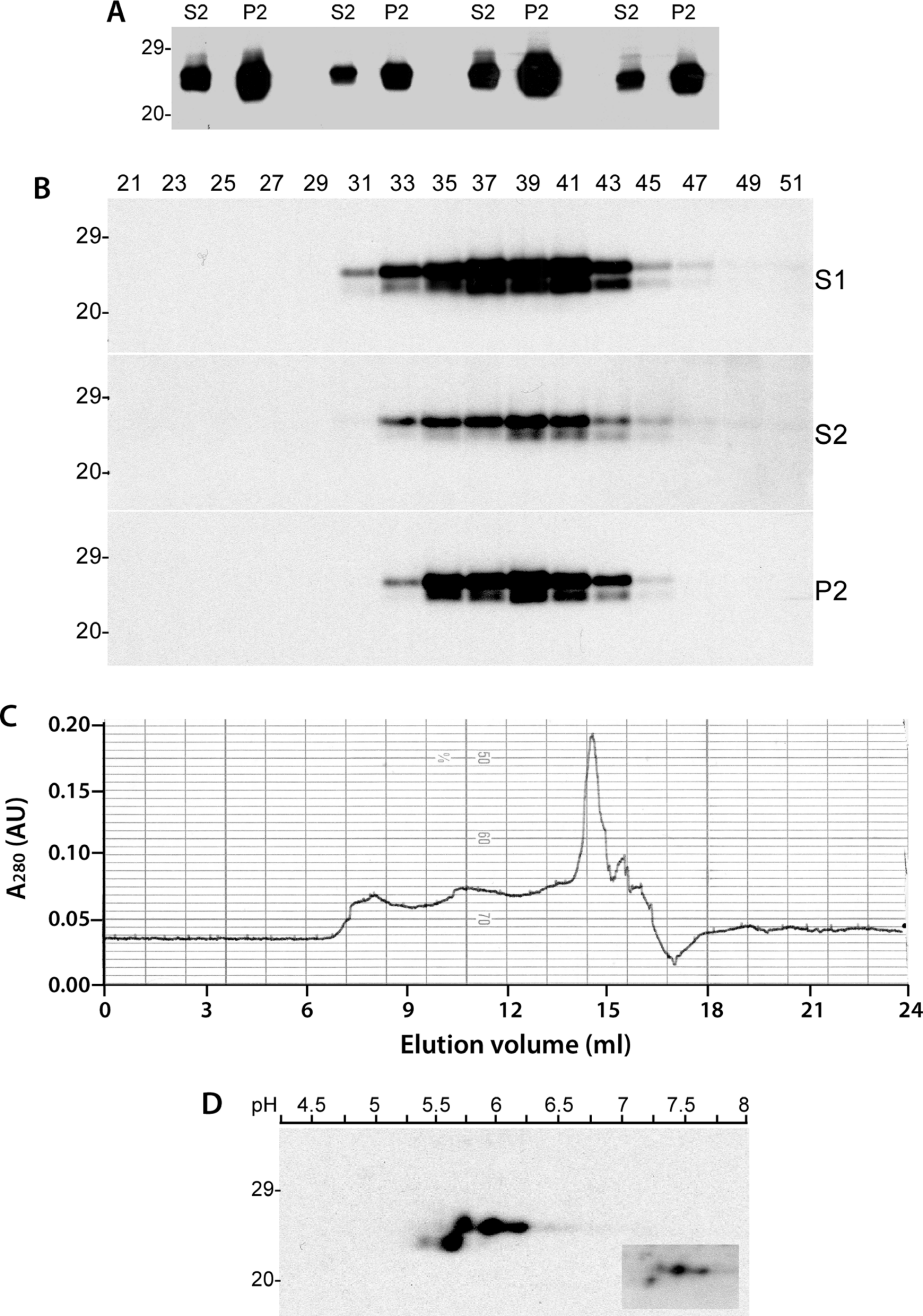
Detergent-soluble and detergent-insoluble caveolin-1 proteins from the human brain **A**. Western blotting of caveolin-1 in both detergent-soluble and- insoluble fractions probed with anti-caveolin-1 antibody. **B**. Western blotting of total caveolin-1 (S1), detergent-soluble (S2) and detergent-insoluble (P2) fractions from size exclusion chromatography (FPLC) probed with anti-caveolin-1 antibody. **C**. Chromatogram of size exclusion chromatography of human brain homogenate from a non-CJD patient. **D**. 2D electrophoresis analysis of caveolin-1 from insoluble fraction. The caveolin-1 recovered in soluble fraction is shown in the insert box. The above Western blots are representative of three independent experiments.

To characterize physicochemical properties of caveolin-1 from the human brain, we next examined detergent solubility of the protein. We observed that caveolin-1 was recovered in both detergent-soluble and -insoluble fractions (Figure 2A). Moreover, the amounts of caveolin-1 were approximately 2-fold higher in the detergent-insoluble fraction than in the soluble fraction based on densitometric analysis of protein bands. We termed caveolin-1 in detergent-soluble fraction as soluble caveolin-1 and the one in detergent-insoluble fraction as insoluble caveolin-1. Using gel filtration with human brain homogenate, we further determined the size of soluble and insoluble caveolin-1 molecules. Surprisingly, there were no significant differences in the molecular mass of caveolin-1 between the two isoforms (Figure 2B and 2C). Most soluble and insoluble caveolin-1 molecules were eluted in the fractions between fractions 33 and 43, corresponding to the molecular markers between 2,000 kDa (fraction 34) and 443 kDa (fraction 44) (Figure 2B). Small amounts of caveolin-1 were also detected in fractions early than 31 (greater than 2,000 kDa) and later than 49 (smaller than 200 kDa) (Figure 2B). The soluble and insoluble caveolin-1 molecules were also evaluated using two-dimensional gel electrophoresis. There were four predominant isoforms that were identified according to their different molecular weight and charges (Figure 2D). Three isoforms migrating at 24 kDa were of molecular charges between *p*I 5.7, 5.8 and 6.2, respectively, which may correspond to α-form of caveolin-1. The fourth isoform migrating at about 22 kDa was of molecular charge at *p*I 5.6, corresponding to β-caveolin-1. Again, although the amounts of various isoforms of caveolin-1 in insoluble fraction were about 2-fold higher than those of various isoforms of caveolin-1 in soluble fraction, the pattern of their distribution was very similar (Figure 2D, insert).

### Caveolin-1 from human brains is composed of both protease-sensitive and resistant domains

**Figure 3:**
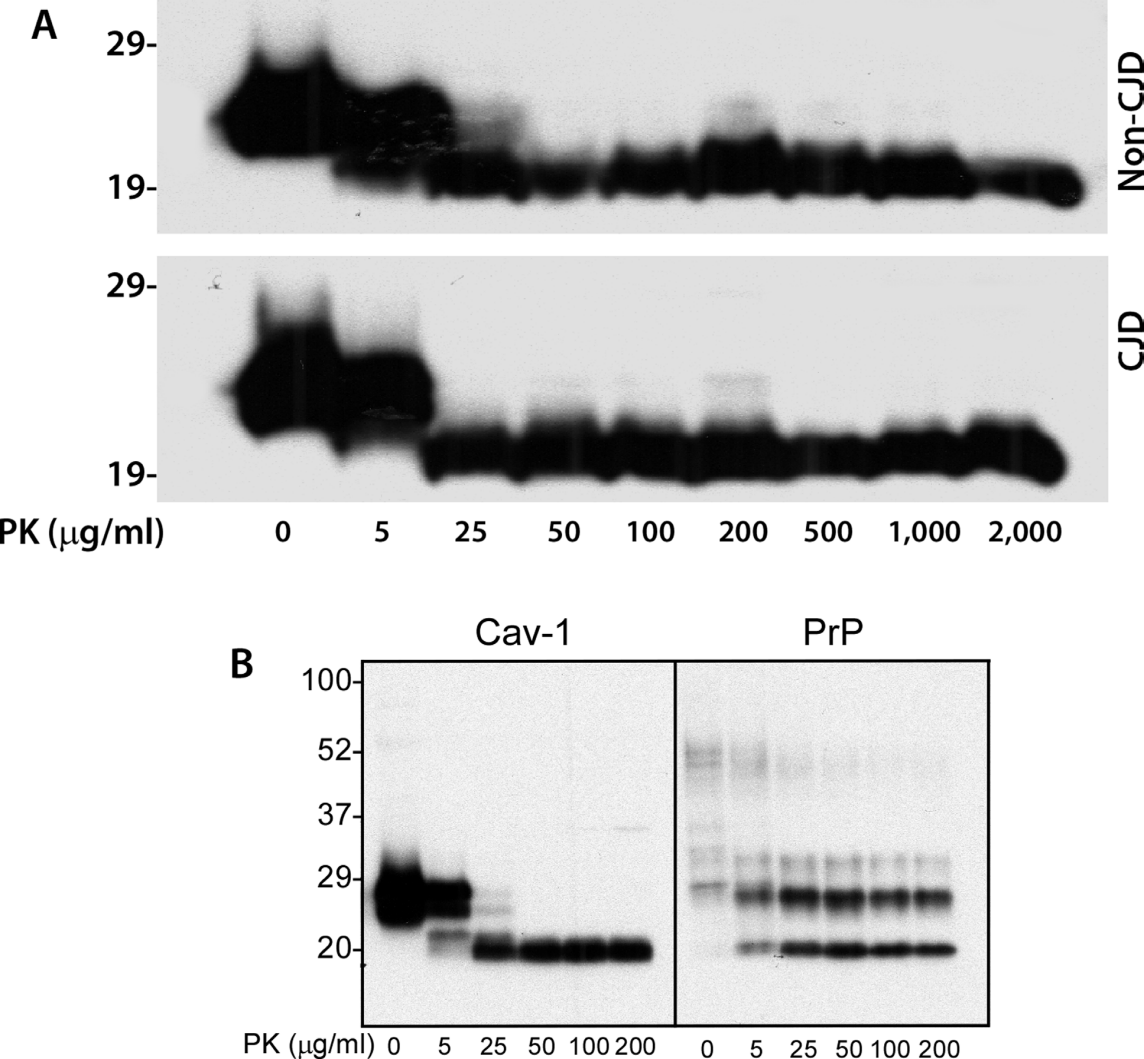
PK-resistance of caveolin-1 from human brain homogenate **A**. Western blotting of brain homogenates from the frontal cortex of CJD and non-CJD patients treated with PK at different concentrations ranging from 0 to 2,000 μg/ml probing with anti-caveolin-1 antibody. **B**. Brain homogenate from a sCJD patient treated with different amounts of PK probing with anti-caveolin-1 or anti-PrP antibody 3F4. The above Western blots are representative of three independent experiments.

It has been believed that most aggregated proteins are resistant to protease digestion, such as the pathological prion protein termed PrP^Sc^ that is associated with various prion diseases including sCJD. To determine if the caveolin-1 oligomers also possess this property, we treated the brain homogenates with proteinase K (PK) at a variety of concentrations from 0 to 2,000 μg/ml for 1 h at 37°C. Compared to untreated sample, the protein migrated faster at ˜21 kDa in the sample treated with 5 μg/ml while the protein in the samples treated with PK from 25 - 2,000 μg/ml migrated consistently at 20 kDa (Figure 3A). The change in molecular weight of caveolin-1 caused by PK-treatment suggests that part of the caveolin-1 molecule is removed by the PK-digestion due to PK-sensitivity while other part is resistant to PK-digestion, a phenomenon called partial PK-resistance characteristic of the PrP^Sc^ molecule [6]. There was no difference in the PK-resistance of caveolin-1 between non-CJD and CJD brain samples (Figure 3A). We also directly compared the PK-resistance between caveolin-1 and PrP^Sc^ from CJD brain tissue homogenate prepared in 1X lysis buffer. Both caveolin-1 and PrP^Sc^ showed a similar PK-resistance: PK-resistant caveline-1 started to be detectable at PK concentration of 5 μg/ml and remained at 200 μg/ml as did PrP^Sc^ (Figure 3B).

### Detergent-soluble and detergent-insoluble caveolin-1 molecules from human brains share a similar resistance to PK-digestion

**Figure 4:**
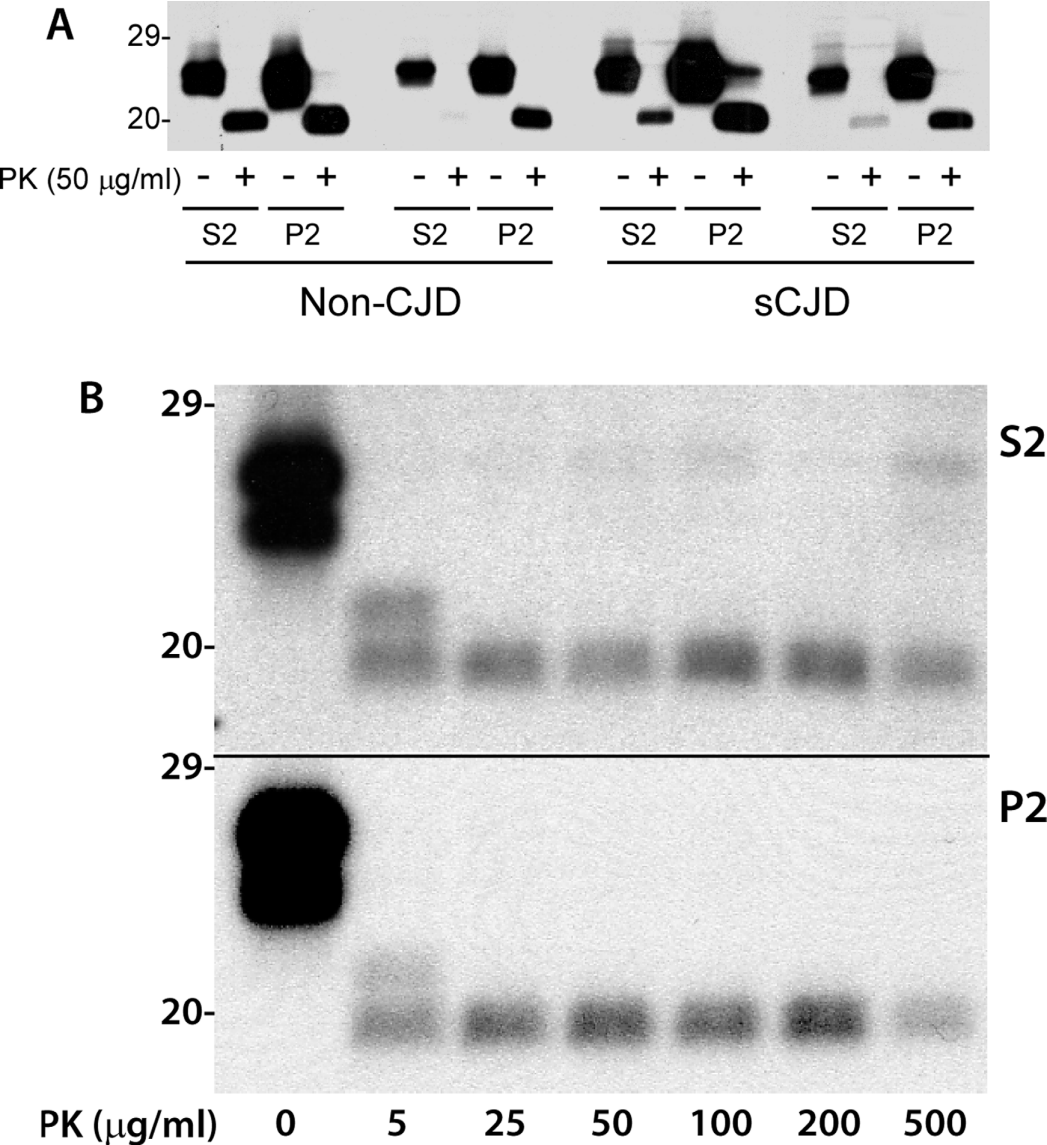
PK-resistant caveolin-1 core fragments in both soluble and insoluble fractions **A**. Western blotting of detergent-soluble (S2) and detergent-insoluble (P2) caveolin-1 molecules after PK-treatment in sCJD and non-CJD cases probing with anti-caveolin-1 antibody. The untreated caveolin-1 migrated between 23 and 24 kDa and PK-treated one migrated between 19 and 20 kDa. **B**. The α- and β-isoforms of caveolin-1 from both S2 and P2 fractions migrated between 22 and 24 kDa without PK treatment, two bands migrating between ˜19 and 21 KDa treated with PK at 5 μg/ml, only one band migrating at ˜19-20 KDa was detectable in the samples treated with PK at 25 μg/ml or higher. The above Western blots are representative of three independent experiments.

It has been well demonstrated that most insoluble PrP is PK-resistant while most soluble PrP is PK-sensitive [7]. We determined the PK-resistance of soluble and insoluble caveolin-1 molecules. Surprisingly, some soluble caveolin-1 molecules were also partially PK-resistant as insoluble caveolin-1 (Figure 4A). To further determine if there are any differences in the resistance to PK-digestion between the two fractions, we treated the detergent-soluble and detergent-insoluble fractions of brain homogenates with various concentrations of PK ranging from 5 μg/ml to 500 μg/ml prior to SDS-PAGE. Without PK-treatment, the α- and β-isoforms of caveolin-1 from both soluble and insoluble fractions migrated between 22 and 24 kDa. Both soluble and insoluble fractions treated with 5 μg/ml showed two bands migrating between ˜19 and 21 kDa (Figure 4B). But only one band migrating at 19-20 kDa was detectable in the samples treated with PK at 25 μg/ml or higher. No significant difference was observed in the resistance to PK digestion between the soluble and insoluble isoforms.

### The amino-terminus of caveolin-1 is sensitive to PK-digestion while its COOH-terminus is PK-resistant

**Figure 5:**
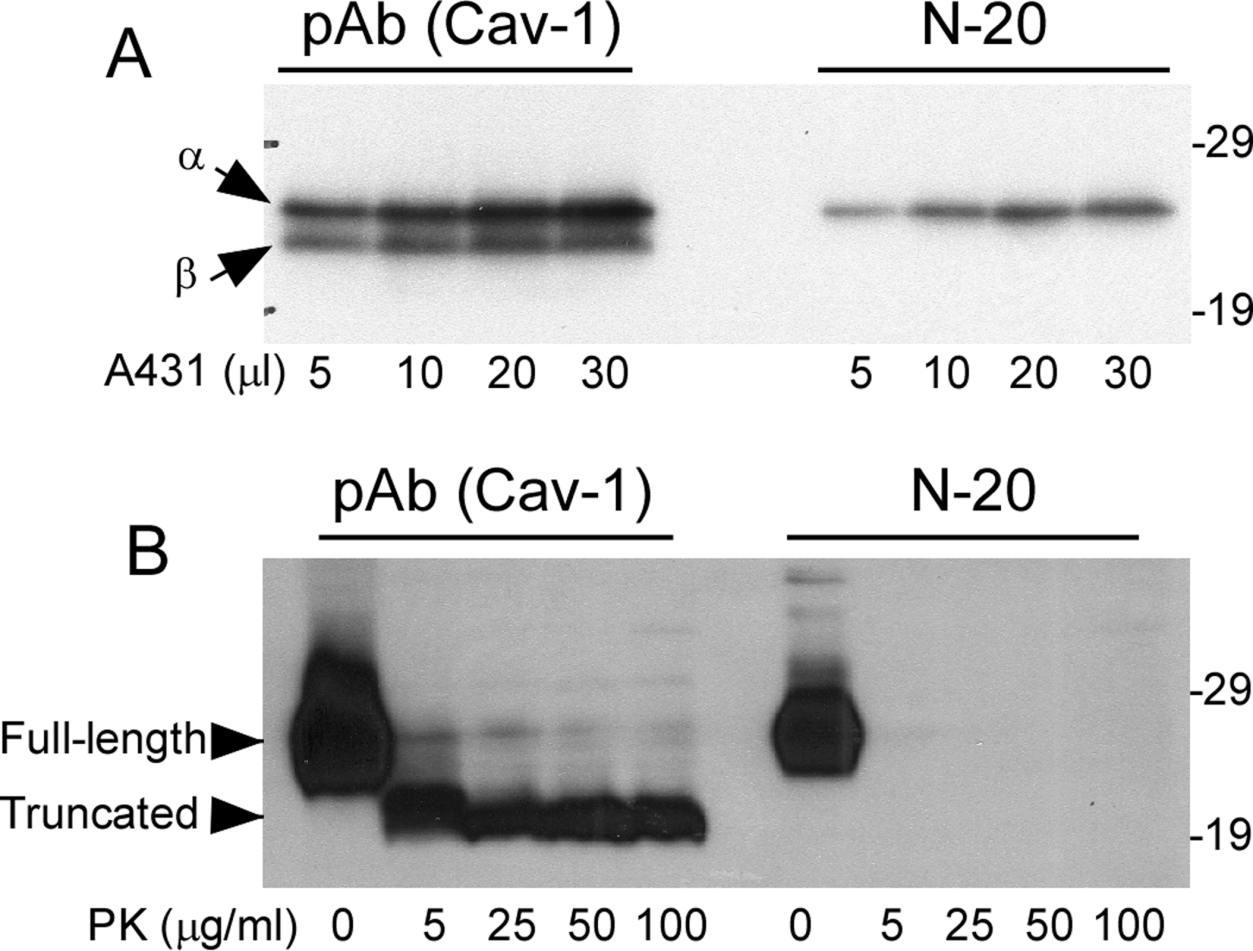
Comparison of PK-sensitive and PK-resistant domains of Caveolin-1 detected with two anti-caveolin-1 antibodies Both pAb (Cav-1) against residues1-97 and Cav-1 (N-20) against residues 1-20 detected caveolin-1 in **A**. A431 cell line and **B**. human brain tissues. Arrows represent α- and β-isoforms of caveolin-1, respectively, in **A**. Arrow heads represent full-length or truncated forms in **B**. The above Western blots are representative of three independent experiments.

To further dissect the molecular structures of caveolin-1 that correspond to PK-sensitivity or PK-resistance, two anti-caveolin-1 antibodies were used against different domains of the protein- pAb (Cav-1) against caveolin-1 residues 1-97 and another called N-20 against caveolin-1 residues 1-20. It has been shown that caveolin-1 from the A431 cell line has two isoforms, a full length α and truncated β form which lacks 31 amino acids from its N-terminal domain [8]. pAb (Cav-1) antibody detected both α- and β- isoforms while N-20 antibody detected the α-isoform only (Figure 5A). When the untreated and treated brain homogenates were probed with the two antibodies, both full-length and truncated fragment were detected with pAb (Cav-1) and only full-length caveolin-1 was detected with N-20 (Figure 5B). This result indicated that the N-terminal domain of caveolin-1 is PK-sensitive, and is removed by the PK-treatment. Since caveolin-1 in the treated sample was lower by about 4 kDa than that in the untreated sample, it is expected that PK may remove about 36 amino acids from the N-terminus of the protein.

### The C-terminus of caveolin-1 from human brains was concealed

**Figure 6:**
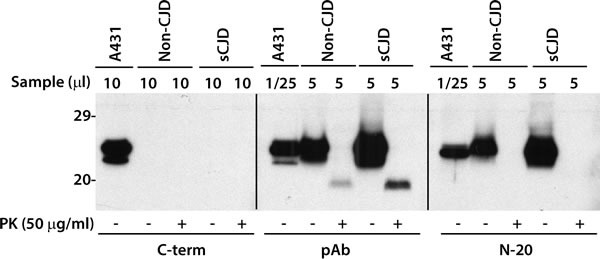
Antibody mapping of caveolin-1 from cultured cells or human brains Caveolin-1 from cultured A431 cells or human brain tissues was treated with or without PK prior to Western blotting probed with three different antibodies against caveolin-1. pAb (Cav-1) against residues1-97 and Cav-1 (N-20) against residues 1-20 detected caveolin-1 while antibody C-term that against C-term region of caveolin only detected caveolin-1 from the cultured cells but not from the brains. The above Western blots are representative of three independent experiments.

Anti-caveolin-1 antibody C-term is an antibody raised against residues 167-178 of the C-terminal domain of caveolin-1. This antibody has been demonstrated to be able to recognize both α- and β-isoforms of caveolin-1 from A431 and NIH 3T3 cell lines. Surprisingly, we found this C-term antibody was unable to recognize any isoform of caveolin-1 from brain samples, whereas it did detect two isoforms of caveolin-1 in A431 cell lysate (Figure 6). The protein from A431 cells migrated at ˜24-25 kDa when probed with the C-term antibody, a gel mobility similar to the protein from the non-CJD and sCJD brains when probed with antibodies pAb and N-20 (Figure 6), suggesting that it is the full-length caveolin-1 that should contain the C-term antibody epitope region. But somehow, the epitope of C-term is hidden or concealed on the caveolin-1 molecules from the brain but not from the cells.

## DISCUSSION

Our current study has made the following observations. First, caveolin-1 is widely present in a variety of human brain regions. Second, the protein in the brain has both detergent-soluble and -insoluble forms. Third, it is partially PK-resistant and is composed of PK-sensitive and PK-resistant domains. Its amino-terminus is PK-sensitive, whereas its COOH-terminus is PK-resistant. Fourth, the soluble and insoluble forms of the protein share a similar resistance to PK digestion. Fifth, soluble or insoluble form is composed of both single molecules and oligomers with molecular mass from 443 kDa and 2,000 kDa. Sixth, the C-terminus of caveolin-1 from human brains is concealed. Finally, no significant differences in oligomerization, solubility in detergents, and resistance to PK of caveolin-1 were observed between normal and prion-infected human brains. The PrP^Sc^-like physicochemical properties of human brain caveolin-1 may be associated with variable structures and functions of caveolin-1.

Caveolin-1 is the integral membrane protein of caveolae and is believed to play roles in lipid transport, membrane traffic, and cell signaling [9, 10]. Its multiple physiological functions may be attributable to the different domain compositions and various intracellular trafficking of the protein. For instance, after synthesis in the endoplasmic reticulum (ER), caveolin-1 is first co-translationally inserted into the ER membrane with its N- and C-terminals exposed to the cytoplasm. Through the involvement of the domain from residues 66-70, caveolin-1 is then transferred to the Golgi apparatus *via* incorporation vesicles [9] and interaction with lipid domains that sort molecules for shipment to the cell surface [10, 11]. In the brain, caveolin-1 has been found in virtually all cell types including endothelial cells, astrocytes, and neurons. Our study revealed that caveolin-1 is highly expressed in the human brain and that the readily detectable amounts of caveolin-1 are widely distributed in different brain areas examined by Western blotting, consistent with previous observations in rat brain [12].

Oligomerization has been found to be an inherent structural feature of caveolin-1 and begins in the ER shortly after synthesis [13]. The tendency to form heptameric oligomers is fundamental to the formation of caveolae filament coats [14]. Based on the studies with recombinant protein, this structural property is associated with an N-terminal α-helical domain between residues 79 and 96, adjacent to a hydrophobic region that is inserted into the cell membrane [14]. This area, termed the caveolin-1 scaffolding domain [15], has been hypothesized to function in inactivation of molecular signaling [16]; however, stimulating or neutral activity has also been observed [17, 18]. Additionally, amino acid residues 91-100 and 134-154 have also been found to mediate oligomerization [9]. Moreover, it has been proposed that a region between residues 66 and 70 is responsible for exit from ER with following region between residues 71 and 80 having a role in transport of caveolin-1 oligomers into detergent-resistant regions of the Golgi apparatus [9]. It would be very interesting to determine in the future whether a prion-like spread mechanism is involved in the oligomerization event of caveolin.

Our study revealed that caveolin-1 exists in a majority oligomeric form in the human brain and that these oligomers are present not only in a detergent-insoluble form, but also in a detergent-soluble form. We observed that approximately two thirds of caveolin-1 are insoluble while another one thirds is soluble. This distribution pattern may depend on the function and localization of oligomers in the cell. Caveolin-1 acquires detergent-insolubility within the Golgi apparatus [19]. The detergent-insoluble caveolin-1 oligomers notably participate in the formation of filament coats of caveola on the cell surface. The detergent-soluble form of caveolin-1 is embedded in lipoprotein-like particles and has been observed in more diverse areas of the cell [10]; for instance, in the lumen of the ER after exposure to cholesterol oxidase [11], and in the cytosol complexed with chaperone proteins [20]. Soluble caveolin-1 oligomers have also been found in the lumen of secretory vesicles and mitochondria [21, 22].

Like the infectious prion protein (PrP^Sc^) and insoluble PrP^C^, caveolin-1 is also partially PK-resistant. Our results revealed that the first N-terminal part of the protein is PK-sensitive. Based on the changes in migration of untreated and PK-treated proteins on Western blots, we estimate that approximately 36 amino acids are removed from the N-terminus of the protein upon PK-treatment. Interestingly, the portion of the protein digested by PK is virtually similar for both soluble and insoluble caveolin-1. In contrast, soluble PrP is PK-sensitive while insoluble PrP is PK-resistant. It is unclear at present whether the PK-resistance results from the protein structure or from other molecules that bound to caveolin-1. Comparison of the molecular basis of PK-sensitivity and detergent-solubility between PrP^Sc^ and caveolin-1 warrants further investigation.

PrP^C^ has been observed to co-localize with caveolin-1 and to participate in signal transduction events by recruiting Fyn kinase [23–25]. However, in prion disease it converts into PrP^Sc^ through a structural transition followed by changes in physicochemical properties. Our current study revealed no significant differences in the amounts and physicochemical properties of caveolin-1 between normal and prion-infected human brains. Whether these results imply the possibility that caveolin-1 and caveolae may not play important roles in the conversion of PrP^C^ into PrP^Sc^ remains to be further studied in the future. Moreover, it is believed that caveolin-1 is mainly present in endothelial cells but not in neurons. Indeed, no significant amounts of caveolin-1 were detected in human neuroblastoma cells by Western blotting (not shown). Therefore, neuronal loss in prion diseases may not be sufficient to cause changes in the levels of caveolin-1. Nevertheless, the possibility cannot be excluded that some of clinical symptoms or signs seen in the early or terminal stage of prion diseases may be associated with dysfunctions of caveolin-1 involved signal transduction due to loss of function of PrP^C^. Indeed, it has been reported that a PrP^C^-caveolin-lyn complex negatively controls neuronal GSK3β and serotonin 1 [24].

## MATERIALS AND METHODS

### Reagents and antibodies

Proteinase K (PK) and phenylmethylsulfonyl fluoride (PMSF) were purchased from Sigma Chemical Co. (St. Louis, MO). Urea, 3((3-Cholamidopropyl) dimethylammonio)-propanesulfonic acid (CHAPS), DL-dithiothreitol (DTT), Iodoacetamide (IAA), tributylphosphine (TBP), Ampholine pH 3-10, and immobilized pH gradient (IPG) strips (pH 3-10, 11 cm long), antibody stripping solution were from Bio-Rad (Richmond, CA). Reagents for enhanced chemiluminescence (ECL Plus) were from Amersham Pharmacia Biotech, Inc. (Piscataway, NJ). Anti-PrP antibody 3F4 (PrP107-112) [26], Rabbit anti-caveolin-1 polyclonal antibody Cav-1 (pAb) against caveolin-1 residues 1-97 (BD Biosciences), rabbit monoclonal antibody Cav-1 (N-20) against caveolin-1 residues1-20 (Epitomics), and C-term against caveolin-1 residues 167-178 (antibodies-online Inc, Atlanta, GA) were used.

### Preparation of brain homogenate and detergent -soluble (S2) and detergent-insoluble (P2) fractions

The 10% (w/v) brain homogenates were prepared in 9 volumes of lysis buffer [10 mM Tris, 150 mM NaCl, 0.5% Nonidet P-40 (NP-40), 0.5% deoxycholate, 5 mM ethylenediaminetetraacetic acid (EDTA), pH 7.4] on ice using pestles with microtubes driven by a cordless motor. When required, brain homogenates were centrifuged at 1,000 x g for 10 min at 4°C to collect supernatant (S1). In order to prepare S2 and P2 fractions, S1 were further centrifuged at 35,000 rpm (100,000 x g) in an SW55 rotor (Beckman Coulter, Fullerton, CA) for 1 h at 4°C. After ultracentrifugation, the supernatants that contain the detergent-soluble fraction were transferred into a clean tube. After being washed gently with 1X lysis buffer twice to remove residual supernatant proteins, the pellets that contain detergent-insoluble fraction (P2) were further re-suspended in the lysis buffer as described [27]. A431 cell lysates were from Epitomics.

### Velocity sedimentation in sucrose step gradients

Supernatant prepared by centrifugation of 20% brain homogenate at 1,000 x g for 10 min at 4°C was incubated with an equal volume of 2% Sarkosyl for 30 min on ice. The sample was loaded atop 10-60% step sucrose gradients and centrifuged at 200,000 x g in the SW55 rotor for 1 h at 4°C as described with minor modification [7, 28, 29]. After centrifugation, the contents of the centrifuge tubes were sequentially removed from the top to the bottom to collect 12 fractions. Aliquots of 12 fractions were subjected to immunoblot analysis described below.

### Size exclusion chromatography

Superdex 200 HR beads (Pharmacia, Uppsala, Sweden) in a 1 × 30 cm column were used to determine the oligomeric state of caveolin-1 molecules. Chromatography was performed in an FPLC system (Pharmacia, Uppsala, Sweden) at a flow rate of 0.25 ml/min and fractions of 0.25 ml each were collected as described [7, 28]. In brief, 200 μl samples, prepared as described above (sucrose step gradients), were injected into the column for each size exclusion run. The molecular weight (MW) of the various PrP species recovered in different FPLC fractions was evaluated according to a calibration curve generated with the gel filtration of molecular mass markers (Sigma, St. Louis, MO) including Dextran blue (2,000 kDa), thyroglobulin (669 kDa), apoferritin (443 kDa), β-amylase (200 kDa), alcohol dehydrogenase (150 kDa), albumin (66 kDa), and carbonic anhydrase (29 kDa). These standards were loaded independently at the concentrations recommended by Sigma in 200 μl sample volumes. The elution volume of blue dextran was used to determine the void volume (V_0_ = 8.45 ml) and the total volume (V_t_ = 24 ml) was provided by the product instruction. The peak elution volumes (V_e_) were calculated from the chromatogram and fractional retentions. Kav were calculated using the equations: K_av_ = (V_e_ - V_0_)/(V_t_ - V_0_) (7). The calibration curve was determined by plotting the Kav of the protein standards against the log MW of the standards (7).

### One- and two- dimensional gel electrophoresis and immunoblotting

Samples were resolved either on 15% Tris-HCl Criterion pre-cast gels (Bio-Rad) for one-dimensional (1D) gel electrophoresis or IPG strips for the two-dimensional (2D) gel electrophoresis. 2D gel electrophoresis was performed as described by the supplier using the PROTEIN IEF cell (Bio-Rad) [7, 30]. Samples denatured by boiling in SDS sample buffer were incubated with reducing buffer (8 M urea, 2% CHAPS, 5 mM TBP, 20 mM Tris-HCl, pH 8.0) for 1 h at room temperature and then incubated with 200 mM IAA for 1 h. Proteins were precipitated with a 5-fold volume of pre-chilled methanol at -20 °C for 2 h and centrifuged at 16,000 x g for 20 min at 4 °C. The pellets were re-suspended in 200 μl of rehydration buffer (7 M urea, 2 M thiourea, 1% DTT, 1% CHAPS, 1% Triton X-100, 1% ampholine pH 3-10, and trace amounts of bromophenol blue). The pellets were dissolved in rehydration buffer and subsequently incubated with the IPG strips for 14 h at room temperature with gentle shaking. The dehydrated gel strips were transferred onto a focusing tray and focused for about 40 kVh. The focused IPG strips were equilibrated for 15 min in equilibration buffer 1 (6 M urea, 2% SDS, 20% glycerol, 130 mM DTT, 375 mM Tris-HCl, pH 8.8), and then another 15 min in equilibration buffer 2 (6 M urea, 2% SDS, 20% glycerol, 135 mM IAA, 375 mM Tris-HCl, pH 8.8). The equilibrated strips were loaded onto the 8-16% Tris-HCl Criterion pre-cast gel (Bio-Rad).

The proteins on the gels were transferred to Immobilon-P membrane polyvinylidene fluoride (PVDF, Millipore) for 2 h at 70V. For probing of the PrP or caveolin-1, anti-PrP antibody 3F4 (PrP107-112), Rabbit anti-caveolin-1 polyclonal antibody pAb (Cav-1) against caveolin-1 residues 1-97 (BD Biosciences), rabbit monoclonal antibody Cav-1 (N-20) against caveolin-1 residues1-20 (Epitomics), and C-term against caveolin-1 residue 167-178 were used. Following incubation with horseradish peroxidase-conjugated sheep anti-mouse IgG or donkey anti-rabbit IgG at 1:3,000, the PrP or caveolin-1 bands or spots were visualized on Kodak film by ECL Plus as described by the manufacturer.
